# Anchoring Plasmonic Ag@AgCl Nanocrystals onto ZnCo_2_O_4_ Microspheres with Enhanced Visible Photocatalytic Activity

**DOI:** 10.1186/s11671-019-2922-1

**Published:** 2019-03-26

**Authors:** Wenhui Liu, Shuangqi Hu, Ying Wang, Bingbing Zhang, Riya Jin, Lishuang Hu

**Affiliations:** Environmental and Safety Engineering Institute, North University of China, Taiyuan, Shanxi 030051 People’s Republic of China

**Keywords:** Composite, Microspheres, Plasmonic, Photocatalytic degradation

## Abstract

In this work, a comprehensive investigation of the composite Ag@AgCl/ZnCo_2_O_4_ microspheres photocatalyst, prepared by a facile two-step method, is presented, and using complementary characterization tools such as X-ray diffraction (XRD), scanning electron microscopy (SEM), energy dispersive X ray spectroscopy (EDX), transmission electron microscopy (TEM), high-resolution transmission electron microscopy (HR-TEM), selected area electron diffraction (SAED), X-ray photoelectron spectroscopy (XPS), UV-Vis diffuse reflectance spectroscopy (DRS), and Brunauer-Emmett-Teller (BET). Results show that the composite Ag@AgCl/ZnCo_2_O_4_ photocatalyst has good microspheres morphology and high crystalline and its absorption intensity in the whole spectrum range is higher than that of pure ZnCo_2_O_4_. It is observed that the specific surface area of the composite Ag@AgCl/ZnCo_2_O_4_ photocatalyst and the adsorption efficiency of rhodamine B (RhB) increase as a result of deposition of Ag@AgCl. In the Ag@AgCl/ZnCo_2_O_4_ degradation system of RhB, the photocatalytic degradation rate of 0.2Ag@AgCl/ZnCo_2_O_4_ becomes 99.4% within 120 min, and RhB is almost completely degraded. The reaction rate constant of composite 0.2Ag@AgCl/ZnCo_2_O_4_ photocatalyst is found to be 0.01063 min^−1^, which is 1.6 times that of Ag@AgCl and 10 times of the minimum value of ZnCo_2_O_4_. In addition, the radical capture experiment indicates that, in the reaction system of Ag@AgCl/ZnCo_2_O_4_, the main oxidative species of Ag@AgCl/ZnCo_2_O_4_ photocatalyst are superoxide anion (O^·^^−^_2__− 2_) and hole (h^+^) and not hydroxyl radical (·OH). Based on the results, a Z-scheme plasmon photocatalytic mechanism of Ag@AgCl/ZnCo_2_O_4_ composite system is proposed, to elucidate the RhB degradation.

## Background

Environmental problems caused by harmful pollutants in water have become a worldwide problem [1] and call for immediate attention of scientists and technologists [2–4]. Nano-semiconductor photocatalytic degradation of organic pollutants in wastewater by visible light is a fascinating and promising research area because of its high efficiency, potential for environmental protection [5–7], and effective utilization of solar radiation [8, 9]. As one of the most important photocatalytic materials, TiO_2_ has been widely investigated due to its high photocatalytic activity, nontoxicity, low cost, and good chemical stability [10]. However, its practical application is largely inhibited because of its wide band gap (3.2 eV for anatase and 3.0 eV for rutile), which means that it can only utilize ultraviolet light (5% of solar energy) [11]. Therefore, a visible light photocatalytic system (43% of solar energy) with high photocatalytic activity is desirable for the efficient utilization of solar radiation [12, 13].

ZnCo_2_O_4_ belongs to a group of spinel oxides [14] with Zn^2+^ residing in the tetrahedral position and the Co^3+^ staying in the octahedral place [15]. Due to the relatively narrow band gap of 2.67 eV [16] and long range (200–800 nm) light response [17], ZnCo_2_O_4_ could be a suitable candidate for photocatalytic organic pollutant degradation [18]. However, ZnCo_2_O_4_ depicts poor quantum yield owing to the low separation of photo inspired electron-hole pairs and week surface visible light photo absorption. This results in inferior photocatalytic efficiency limiting its practical applicability. In order to overcome these drawbacks, coupling ZnCo_2_O_4_ with other semiconductors could be a good strategy which could lead to improved separation of photo-induced electron and high photocatalytic activity. For example, *Rajakumar Ananthakrishnan* et al. synthesized heterostructured cation-doped ZnO-ZnCo_2_O_4_ nanocomposites and the decolorization rate of methyl orange was found to reach 92% under visible light [19].

Literature shows the study of different Ag@AgCl-based heterogeneous photocatalytic systems such as H_2_WO_4_.H_2_O/Ag/AgCl [20], Ag@AgCl-Bi_2_MoO_6_ [21], Ag@AgCl/WO_3_ [22], and Ag@AgCl/rGO [23]. The bandwidth of AgCl is 3.25 eV, which cannot absorb visible light. Ag@AgCl demonstrates excellent visible light absorption, which comes from the surface plasmon resonance (SPR) effect produced by the metallic Ag on the AgCl surface [24]. The dispersed AgCl can promote photoinduced charge carriers separation efficiency. Both the excellent visible light absorption of Ag@AgCl and enhanced carriers separation can lead to the improvement of photocatalytic activity.

It is apparent from the above analysis that the ZnCo_2_O_4_ photocatalytic activity can evidently be improved by anchoring plasmonic Ag@AgCl nanocrystals onto ZnCo_2_O_4_. Herein, the Ag@AgCl/ZnCo_2_O_4_ composite was prepared with a facile two-step solvothermal method. The composite was characterized by X-ray diffraction (XRD), scanning electron microscopy (SEM), energy dispersive X ray spectroscopy (EDX), transmission electron microscopy (TEM), high-resolution transmission electron microscopy (HR-TEM), selected area electron diffraction (SAED), X-ray photoelectron spectroscopy (XPS), UV-Vis diffuse reflectance spectroscopy (DRS), and Brunauer-Emmett-Teller (BET). The influence of ZnCo_2_O_4_ structural characteristics and absorbance properties before and after loading Ag@AgCl are carefully investigated. The activity and stability of photocatalytic degradation of rhodamine B (RhB) are also presented. A mechanism to enlighten the degradation mechanism of RhB in Ag@AgCl/ZnCo_2_O_4_ photocatalytic system is proposed.

## Methods

### Synthesis of ZnCo_2_O_4_ Microspheres by Microwave-Assisted Method

In a typical synthesis procedure, 2.3 g Zn (NO_3_)_3_.6H_2_O, 4.48 g Co (NO_3_)_3_.6H_2_O, 3.6 g CO (NH_2_)_2_, and 1.14 g NH_4_F were dissolved in 100 mL deionized water with stirring for 30 min and then ultrasonic dispersion 30 min to obtain pink solution. The above pink solution was transferred to a 300 mL polytetrafluoroethylene reactor and then the reactor was connected to the microwave reaction apparatus. The heating rate was set at 8 °C/min and the microwave reacted at 130 °C for 30 min. After the reaction was finished, the reactor was cooled to room temperature. The pale pink precursor was collected via centrifugation, washed three times with deionized water and absolute ethanol respectively to remove the possible residues, then dried at 80 °C for 10 h in oven, and calcined at 350 °C for 2 h in tube muffle furnace at 1 °C/min to obtain the samples.

### Synthesis of Ag@AgCl/ZnCo_2_O_4_ Microspheres

In a typical synthesis of Ag@AgCl/ZnCo_2_O_4_ microspheres, 0.17 g AgNO_3_ was dissolved in 80 mL mixed solvent of alcohol and water with volume ratio of 3:5. Then, 0.2 g ZnCo_2_O_4_ and 0.1 11 g PVP were added to the above mixed solution under magnetic stirring. After heated at 130 °C for 3 h, the Ag^+^-ZnCo_2_O_4_ solution formed. Further, 1.5 g L^−1^ of NaCl aqueous solution (20 mL) was add to the above solution, then the pH was adjusted to about 2.5 with HCl (12 wt%). The solution was stirred for 24 h by avoiding light and magnetic force. Some Ag^+^ in the solution was reduced to Ag by irradiated the solution with 1000 W xenon lamp for 30 min. Ag@AgCl/ZnCo_2_O_4_ catalyst was prepared by centrifugal separation, washed three times with deionized water and anhydrous ethanol respectively, drying at 80 °C for 6 h in oven.

In addition, Ag@AgCl catalyst was prepared without the presence of ZnCo_2_O_4_ with other condition unchanged.

### Characterization

The phase composition of the obtained sample was recorded on a D/MaxRB X-ray diffractometer (Japan) with Cu-Kα radiation source at 35 kV, with a scanning rate of 0.02°s^−1^ in the 2θ range from 10° to 75°. The morphologies were studied by JSM-6510 scanning electron microscopy (SEM) and JSM-2100 transmission electron microscopy (TEM) equipped with an energy dispersive X-ray spectra (EDX). X-ray photoelectron spectroscopy (XPS) data were obtained with an ESCALab220i-XL electron spectrometer from VG Scientific using 300 W AlKα radiation. Base pressure was about 3 × 10^−9^ mbar. The binding energies were referenced to the C1s line at 284.6 eV from amorphous carbon. The BET specific surface area of the samples was investigated by a high-speed automated area and pore size analyzer (3H-2000PS1, China).

### Photocatalytic Activity Measure

The photocatalytic activity of as-prepared Ag@AgCl ZnCo_2_O_4_ microspheres catalysts was evaluated by the photodegradation of rhodamine B (RhB) in aqueous solution. In every experiment, 50 mg catalysts were dispersed in 50 mL of RhB aqueous solution (10 mg L^−1^). Before light irradiation, the suspension was sufficiently stirred in the darkness for 30 min to make sure the adsorption-desorption equilibrium. The temperature of suspensions was kept below 283 K by a flow of cooling water during the reaction and the irradiation was performed with a 1000 W Xenon lamp. The change of RhB concentrations (C) based on the irradiation time was measured by LAMBDA35 ultraviolet/visible spectrophotometer (λ = 553 nm, Perkin Elmer Instruments Co, Ltd., America). As a time function, the decolorization rate is expressed as *C*_*t*_/*C*_0_, where *C*_0_ is the initial concentration of RhB and *C*_t_ is the instantaneous concentration in the solution. The cycle stability of the sample is detected as follows. After the photocatalytic performance, the samples are collected after several washing and drying. Then, four reuse times of the experiment mentioned above was repeated.

## Results and Discussion

**Fig. 1:**
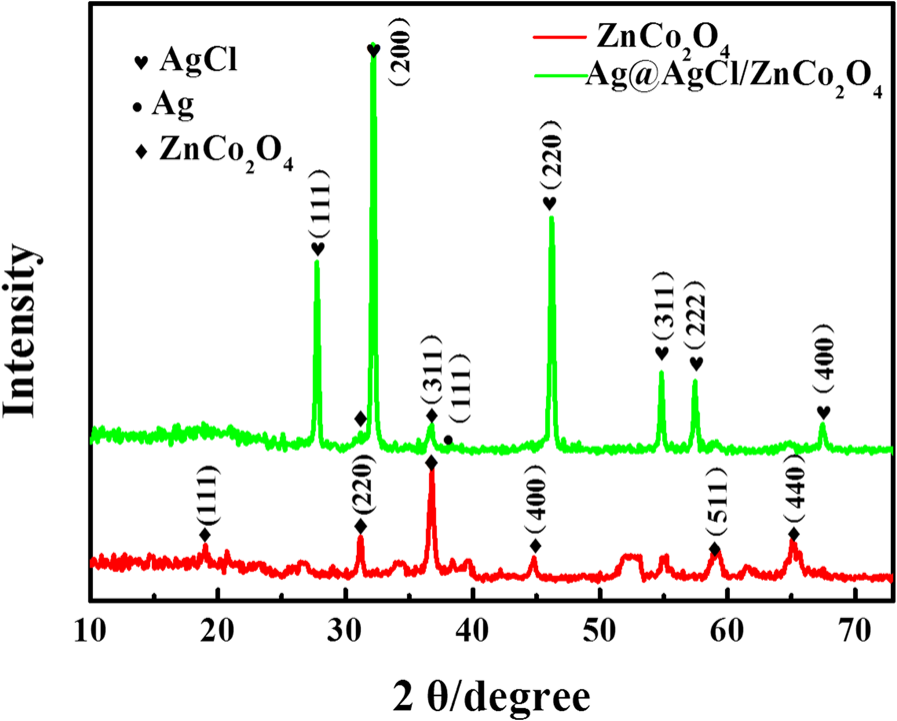
The XRD patterns of the as-prepared ZnCo_2_O_4_ and Ag@AgCl/ZnCo_2_O_4_ microspheres samples

The phase structure and crystal form of the catalysts were determined by XRD. In Fig. 1, the XRD spectra of ZnCo_2_O_4_, Ag@AgCl/ZnCo_2_O_4_ catalysts were shown. The diffraction peaks of ZnCo_2_O_4_ at 18.96°, 31.215°, 36.805°, 44.738°, 59.282°, and 65.149° were observed, corresponding to (111), (220), (311), (400), (511), and (440) crystal faces of the cubic spinel structured ZnCo_2_O_4_ (JCPDS No. 23-1390), respectively, indicating that the ZnCo_2_O_4_ was synthesized by microwave-assisted method. After loading Ag@AgCl, the characteristic diffraction peaks of 27.8°, 32.2°, 46.2°, 54.8°, 57.5°, and 67.5° from Ag@AgCl/ZnCo_2_O_4_ correspond to the (111), (200), (220), (311), (222), and (400) faces of cubic AgCl (JCPDS No. 85-1355). In addition, combined with cubic Ag (JCPDS No. 87-0719), the XRD spectra of Ag@AgCl/ZnCo_2_O_4_ show that there is one characteristic diffraction peak of Ag nanoparticles at 38.2°, indicating the existence of Ag in the catalyst. Because some Ag^+^ is reduced to Ag particles in the process of photo-reduction, which makes the photocatalytic performance of Ag@AgCl/ZnCo_2_O_4_ improved significantly under visible light.

**Fig. 2:**
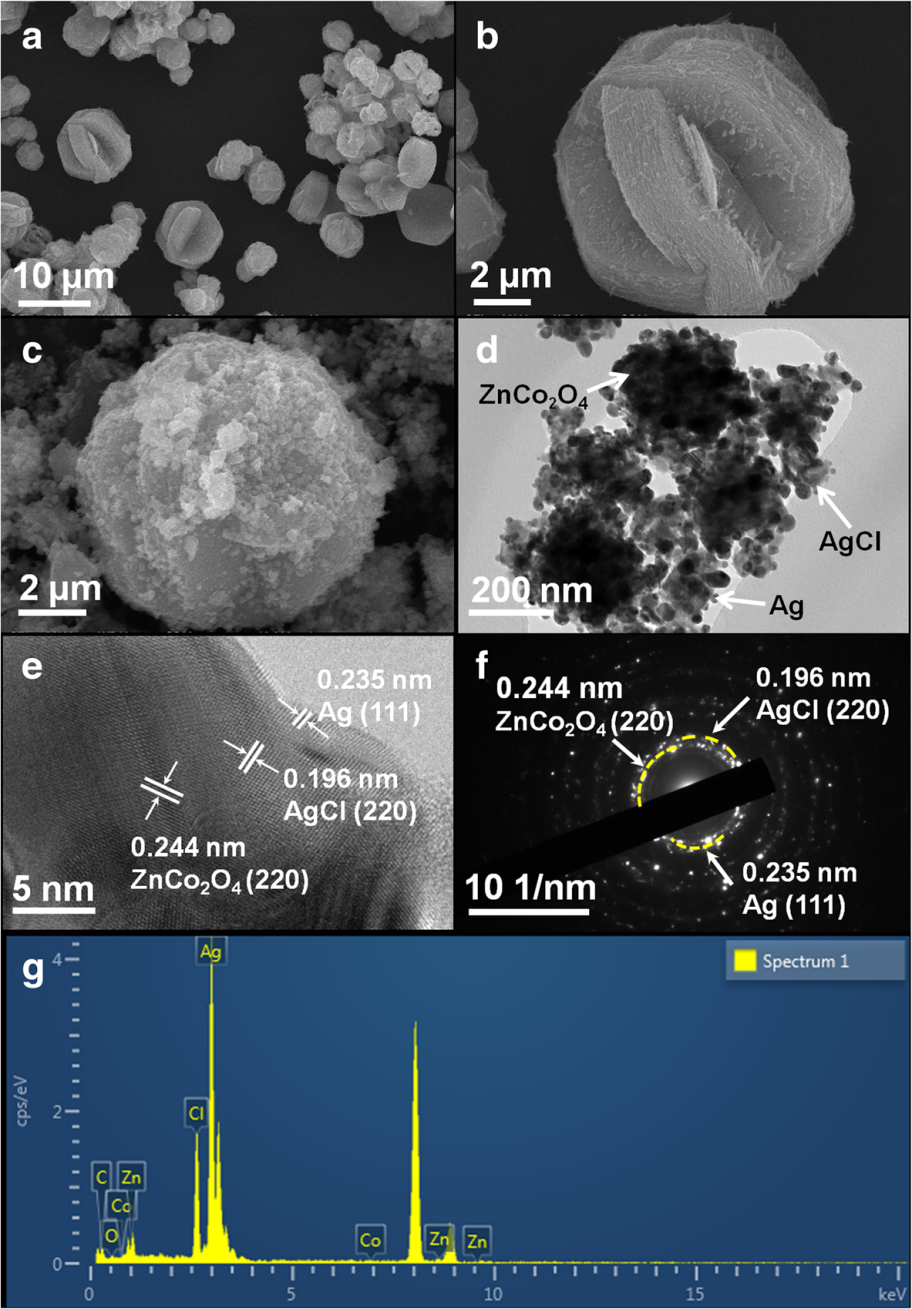
**a**, **b** SEM images of the ZnCo_2_O_4_ microspheres. **c**–**e** SEM, TEM, and HRTEM images of the Ag@AgCl/ZnCo_2_O_4_ microsphere. **f**, **g** SAED and corresponding EDS patterns of the Ag@AgCl/ZnCo_2_O_4_

In Fig. 2a, spherical ZnCo_2_O_4_ microstructures with diameters ranging from 5 to 8 μm were successfully prepared via a microwave-assisted method. ZnCo_2_O_4_ microsphere structure consists of stacked lamellar (Fig. 2b). Figure 2c is the SEM image of Ag@AgCl/ZnCo_2_O_4_ after loading. It can be seen that Ag@AgCl nanocrystals were loaded on the surface of spherical ZnCo_2_O_4_. In order to further observe the morphology of Ag@AgCl/ZnCo_2_O_4_, the TEM image of Ag@AgCl/ZnCo_2_O_4_ is shown in Fig. 2d. From the TEM image, it can be seen that 10–50 nm Ag nanoparticles are uniformly attached to the surface of ZnCo_2_O_4_, and 20–100 nm AgCl particles are dispersed on the surface of ZnCo_2_O_4_. Figure 2e shows the HRTEM of Ag@AgCl/ZnCo_2_O_4_. It can be seen that Ag and AgCl particles are loaded on ZnCo_2_O_4_, and the fringes spacing d of Ag, AgCl, and ZnCo_2_O_4_ are 0.235, 0.196, and 0.244 nm, corresponding to the crystal faces Ag(111), AgCl(220), and ZnCo_2_O_4_(220), respectively. Figure 2f is SAED of Ag@AgCl/ZnCo_2_O_4_. The diffraction ring of Ag@AgCl/ZnCo_2_O_4_ is regular and bright, indicating that it is a polycrystalline with good crystalline. The three crystal planes have a lattice spacing of 0.244 nm, 0.235 nm, and 0.196 nm, which agree well with the HRTEM results. The EDX image of Ag@AgCl/ZnCo_2_O_4_ in Fig. 2g shows that the sample is composed of five elements: O, Co, Zn, Cl, and Ag. The intensity of the peaks in the image represents the content of each elements. Zn, Co, and O are composed of ZnCo_2_O_4_, whereas Ag and Cl are composed of Ag@AgCl. EDX confirmed the chemical elements corresponding to Ag@AgCl/ZnCo_2_O_4_ and did not detect other elements. In conclusion, Ag@AgCl can be clearly determined to be uniformly dispersed and loaded on the surface of ZnCo_2_O_4_ microspheres.

**Fig. 3:**
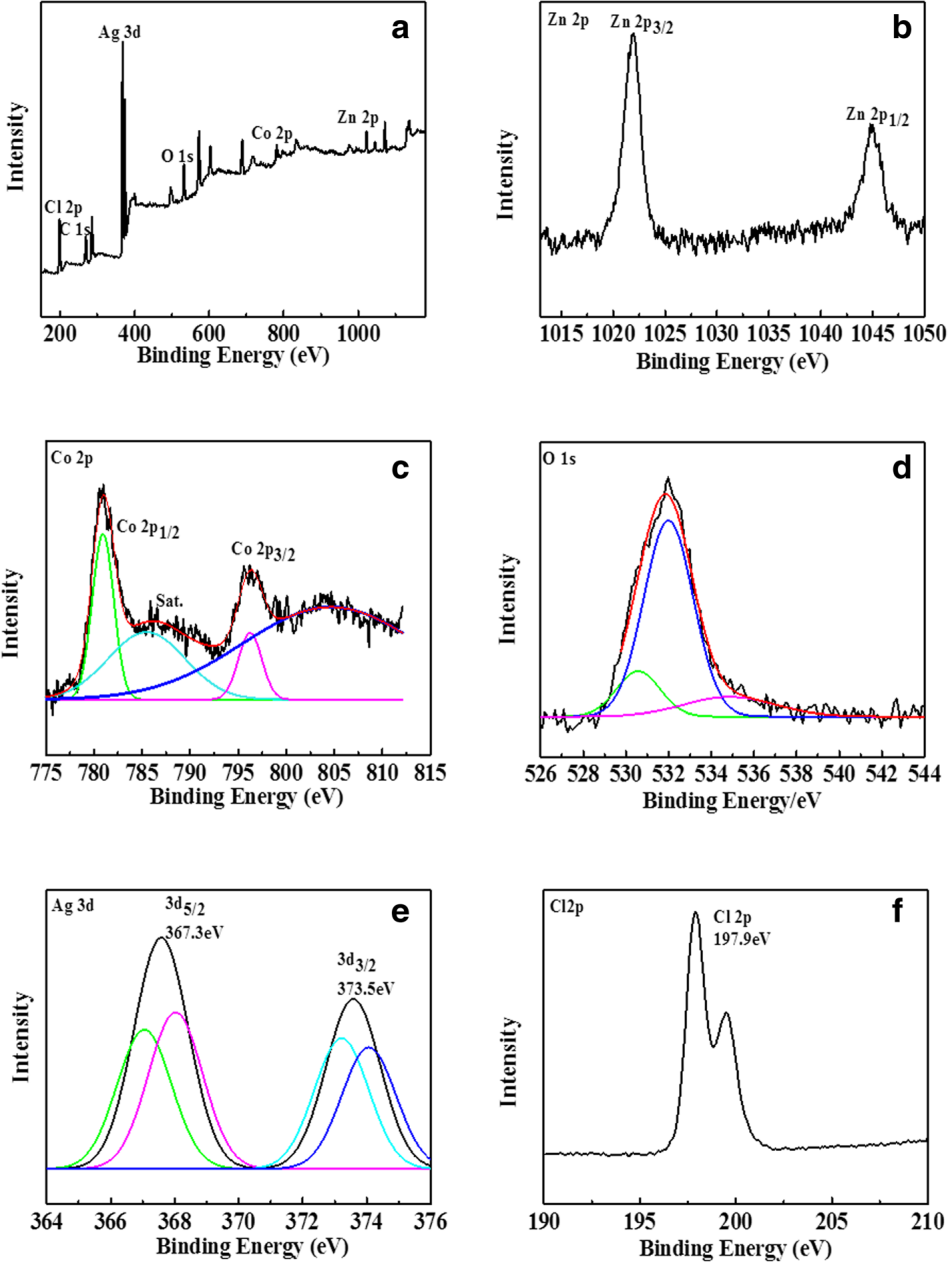
XPS spectrum of Ag@AgCl/ZnCo_2_O_4_: **a** survey scan, **b** Zn 2p, **c** Co 2p, **d** O 1 s, **e** Ag 3d, and **f** Cl 2p

X-ray photoelectron spectroscopy (XPS) was used to determine the composition and chemical valence of the products. As shown in Fig. 3, Fig. 3a is a full spectrum scan of the product. It can be seen that the product contains six elements, namely Zn, Co, O, Ag, Cl, and C, of which C is the base. Figure 3b shows the emission spectra of Zn 2p. Two main peaks appear at 1045 eV and 1022 eV, corresponding to the regional peaks of Zn 2p_1/2_ and Zn 2p_3/2_ [25, 26]. It can be seen that the peak of Zn 2p_3/2_ near 1022 eV is a single peak, which is a typical oxidation state of Zn^2+^. Figure 3c shows the XPS peaks of Co, which correspond to the regional peaks of Co 2p_1/2_ and Co 2p_3/2_ at 781.4 eV and 796.9 eV, and the obvious satellite peaks observed at 785.2 eV are characteristic peaks of Co^3+^ oxidation state [27]. Figure 3d is the XPS spectra of O1s. The asymmetric peaks can be divided into two groups of characteristic peaks with binding energies of 530.5 eV and 535.01 eV, respectively. These two groups of characteristic peaks correspond to the oxygen in the spinel ZnCo_2_O_4_ lattice and the water molecules or ·OH groups adsorbed on the surface of the material [28]. The XPS spectra of Ag 3d orbits are shown in Fig. 3e. The binding energies of Ag 3d at 367.3 eV and 373.5 eV correspond to the spin cleavage orbits of Ag 3d_5/2_ and Ag 3d_3/2_, respectively [29]. The spin splitting orbits of Ag 3d_5/2_ can be further decomposed into 368.0 eV and 366.8 eV peaks by peak splitting software. Similarly, the spin splitting orbits of Ag 3d_3/2_ can be decomposed into 374.0 eV and 372.6 eV peaks, of which 368.0 eV and 374.6 eV belong to Ag^0^, while 366.8 eV and 372.6 eV belong to Ag^+^, indicating that AgCl and Ag are formed in the catalyst. Figure 3f is the XPS analytic diagram of Cl 2p, and the electron binding energy of Cl 2p appears in 197.9 eV.

**Fig. 4:**
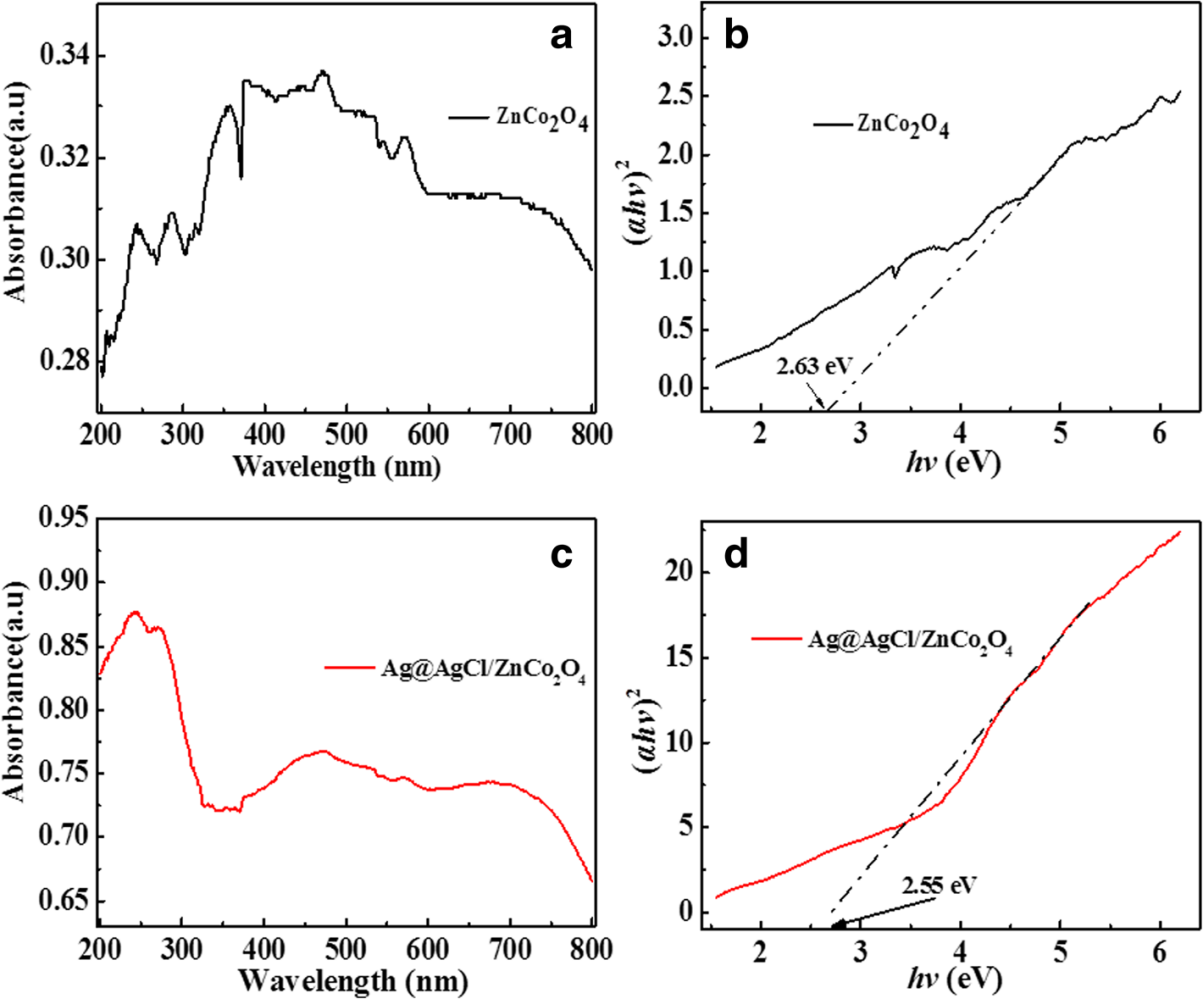
**a** UV-Vis diffuse reflectance spectra of ZnCo_2_O_4_. **b** Plots of (*αhv*)^2^ versus energy (*hv*) for the band gap energy of ZnCo_2_O_4_. **c** UV-Vis diffuse reflectance spectra of 0.2Ag@AgCl/ZnCo_2_O_4_. **d** Plots of (*αhv*)^2^ versus energy (*hv*) for the band gap energy of 0.2Ag@AgCl/ZnCo_2_O_4_

The UV-Vis diffuse reflectance absorption spectra of ZnCo_2_O_4_ and 0.2Ag@AgCl/ZnCo_2_O_4_ catalysts were compared in Fig. 4a, c. The results showed that all the samples exhibited strong absorption in the UV-Vis region, and 0.2Ag@AgCl/ZnCo_2_O_4_ has stronger absorption capacity than ZnCo_2_O_4_. The forbidden band width of ZnCo_2_O_4_ and Ag@AgCl/ZnCo_2_O_4_ catalysts is calculated according to the Kubelka-Munk formula [30]:

$$ \mathrm{A} hv=\mathrm{c}{\left( hv-\mathrm{Eg}\right)}^n $$

Among them, *A* is the absorption coefficient, *h* is the Planck constant, *v* is the frequency of light, *c* is the constant, Eg is the bandgap width, and *n* is the constant coefficient, for direct semiconductors, *n* = 1/2, for indirect semiconductors, *n* = 2.

Figure 4b, d shows the band gap energy (*αhv*)^2^ and energy (*hv*) diagrams of ZnCo_2_O_4_ and Ag@AgCl/ZnCo_2_O_4_ catalysts. The band gap widths are 2.63 eV and 2.55 eV, respectively. Compared with ZnCo_2_O_4_, Ag@AgCl/ZnCo_2_O_4_ catalysts have narrow band gap and are more easily excited by visible light to produce free radicals, so the photocatalytic performance of Ag@AgCl/ZnCo2O4 is expected to be better.

**Fig. 5:**
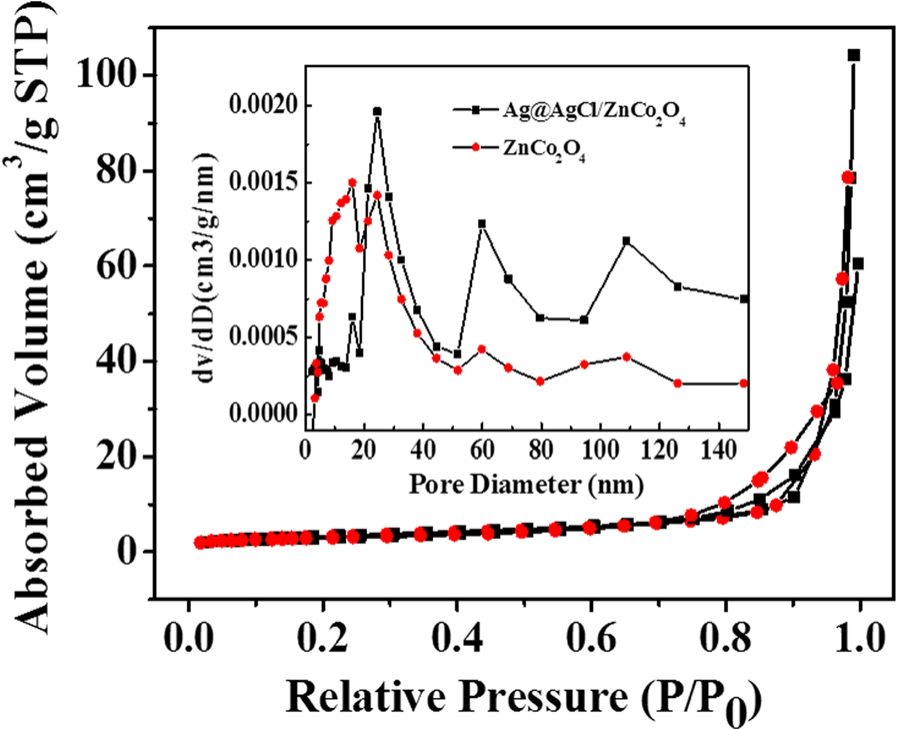
Nitrogen adsorption-desorption isotherms and pore size distribution of ZnCo_2_O_4_ and 0.2Ag@AgCl/ZnCo_2_O_4_ samples

The specific surface area is one of the important factors for the activity of photocatalysts. The specific surface area and pore size distribution of ZnCo_2_O_4_ and 0.2Ag@AgCl/ZnCo_2_O_4_ samples were obtained by N_2_ adsorption-desorption isothermal measurement. The obtained curves are shown in Fig. 5. The N_2_ adsorption-desorption isotherms of the two samples showed obvious hysteresis loops and belonged to type IV isotherms, which proved that the microspheres composed of nanosheets had mesoporous structure. The formation of mesoporous ZnCo_2_O_4_ microspheres was mainly attributed to the voids formed during the self-assembly of nanosheets and the random stacking of nanoparticles during Ag@AgCl loading. The BET specific surface areas of ZnCo_2_O_4_ and Ag@AgCl/ZnCo_2_O_4_ samples were measured by N_2_ adsorption method. The BET specific surface areas of the samples are 9.977 m^2^/g and 11.67 m^2^/g, respectively. The results show that the specific surface area of ZnCo_2_O_4_ microspheres can be increased by loading Ag@AgCl, which is mainly due to the large specific surface area of Ag @AgCl nanoparticles with the diameter of 50–100 nm. Large specific surface area can not only make the material have better adsorption performance but also provide more active sites, and facilitate the transfer of charge carriers, which is helpful to further promote the photocatalytic performance of the material.

In addition, desorption branch curves of the N_2_ adsorption-desorption isotherms of the two samples has been calculated by Barrett-Joyner-Halender (BJH) model. The pore size distribution curves of the samples are shown in the insert in Fig. 5. The pore size distribution curves show that the pore size distribution of ZnCo_2_O_4_ is mainly at 15.96 nm, while that of Ag@AgCl/ZnCo_2_O_4_ is mainly at 24.47 nm. Such pore structure is very conducive to the adsorption of reactants, the transport of products, and capture of photoenergy, thus improving the photocatalytic properties of materials.

**Fig. 6:**
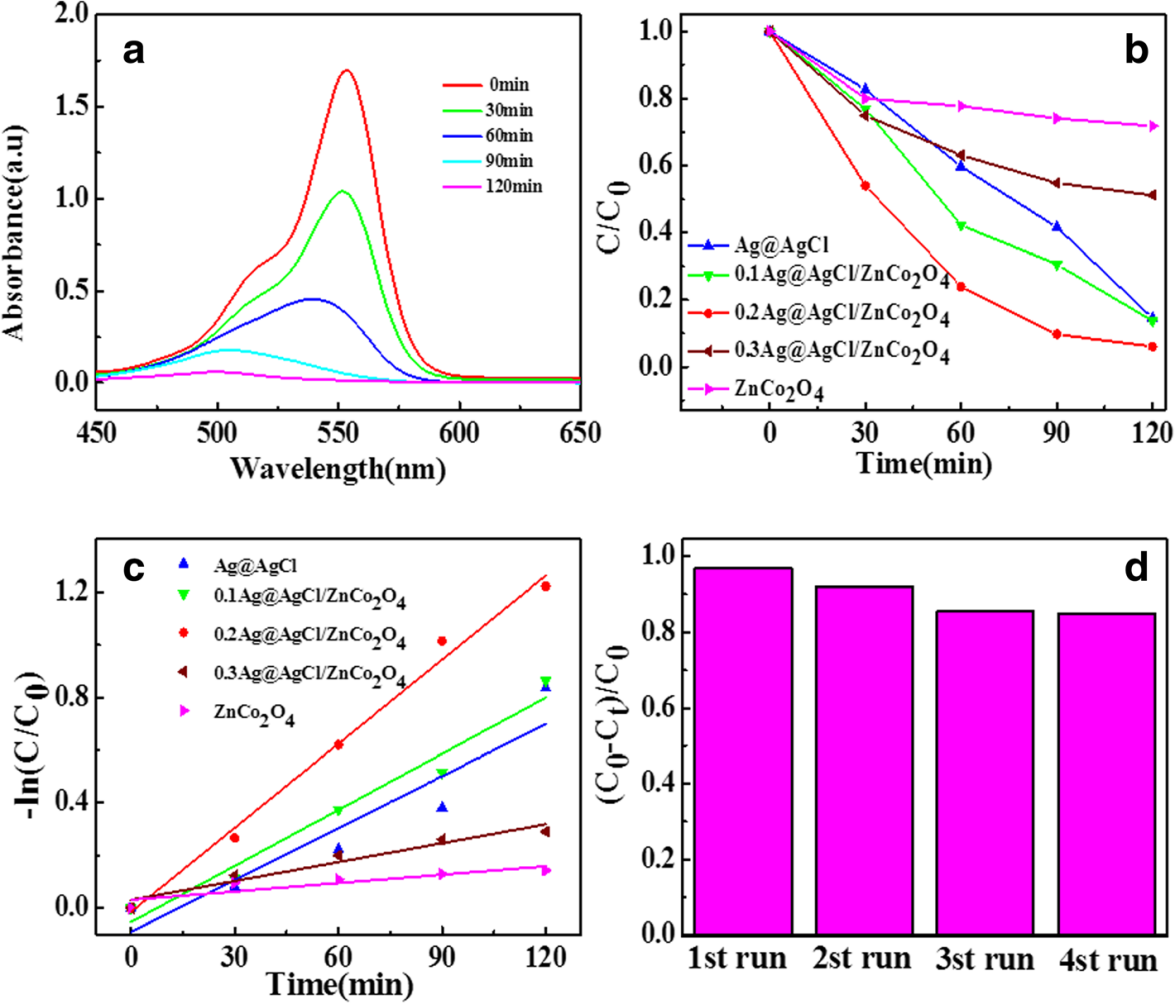
**a** Visible light scanning pattern of 0.2Ag@AgCl/ZnCo_2_O_4_ degradation of RhB. **b** Effects of different catalysts on photocatalytic degradation of RhB under visible light. **c** First-order kinetic fitting plots for degradation of RhB by different catalysts. **d** Cycling runs of 0.2Ag@AgCl/ZnCo_2_O_4_ microspheres for the degradation of RhB

In order to study the photocatalytic activity of the prepared samples, RhB degradation experiments were carried out under visible light. The change of RhB during the photocatalytic degradation of 0.2Ag@AgCl/ZnCo_2_O_4_ was analyzed by UV-Vis full-wavelength scanning. The results are shown in Fig. 6a. The absorption peak of RhB is near 553 nm, which is the characteristic absorption of azo bond in RhB molecule, that is, the chromogenic group of RhB dye molecule. As the reaction time progressed, the peak intensity at 553 nm became lower and lower, which indicated that the chromophore group of RhB was destroyed under the action of photocatalyst. After 120 min of irradiation, the molecule of RhB was completely decolorized, and the peak intensity at 550 nm was almost zero, indicating that the azo structure of RhB dye had been completely destroyed. In addition, during the degradation process, the shape of the absorption peak of RhB molecule changed broad and slight blue shift of the peak appeared, which indicated that some small molecular intermediates were produced during the degradation process.

As shown in Fig. 6b, the photocatalytic degradation process of RhB over ZnCo_2_O_4_, 0.1Ag@AgCl/ZnCo_2_O_4_, 0.2Ag@AgCl/ZnCo_2_O_4_, 0.3Ag@AgCl/ZnCo_2_O_4_, and Ag@AgCl catalysts were investigated. The photocatalytic performances of different catalysts are analyzed and compared. The results indicate that the pure ZnCo_2_O_4_ photocatalytic degradation was the worst and the photocatalytic degradation rate for 120 min was only 28%. The photocatalytic degradation rate of 0.3Ag@AgCl/ZnCo_2_O_4_ in 120 min is 48.8%. The photocatalytic degradation rate of 0.1Ag@AgCl/ZnCo_2_O_4_ in 120 min is seen to be 85.4%, which is very close to the photocatalytic degradation rate of Ag@AgCl 86.3%. The results indicate that within 120 min, 99.4% photocatalytic degradation of 0.2Ag@AgCl/ZnCo_2_O_4_ takes place and RhB is found to be completely degraded. The experimental results show that Ag@AgCl can effectively enhance the photocatalytic degradation performance of ZnCo_2_O_4_ photocatalyst.

In order to study the kinetic model of photocatalytic reaction of different catalysts, Fig. 6c was obtained from equation –ln(C/C_0_) = kt. It is evident from the graph that (*C*/*C*_0_) is linearly correlated with reaction time *t* and *k* is the apparent reaction rate constant, which indicates that the photocatalytic degradation of RhB follows pseudo-first-order kinetic model. As shown in Table 1, the *k* values of each sample were calculated after linear fitting of the curve. As shown in Table 1, the reaction rate constants of ZnCo_2_O_4_, 0.1Ag@AgCl/ZnCo_2_O_4_, 0.2Ag@AgCl/ZnCo_2_O_4_, 0.3Ag@AgCl/ZnCo_2_O_4_, and Ag@AgCl are 0.00107 min^−1^, 0.0071 min^−1^, 0.01063 min^−1^, 0.00239 min^−1^, and 0.00657 min^−1^, respectively. Among them, the reaction rate constant of 0.2Ag@AgCl/ZnCo_2_O_4_ is the largest, 0.01063 min^−1^, 1.6 times of Ag@AgCl and 10 times of the minimum value of ZnCo_2_O_4_. This shows that the composite of Ag@AgCl and ZnCo_2_O_4_ can support Ag@AgCl on the surface of ZnCo_2_O_4_ and promote the dispersion of Ag@AgCl, which can increase the specific surface area of the catalyst and provide more active sites to improve the substrates photocatalytic activity.

**Table 1:** Photodegradation rate constants and linear regression coefficients of different catalysts from equation –ln(C/C_0_) = kt

	K (min^−1^)	Regression equation	*R* ^2^
0.2Ag@AgCl/ZnCo_2_O_4_	0.01063	-ln(C/C_0_) = 0.01063x − 0.01337	*R*^2^ = 0.9894
0.1Ag@AgCl/ZnCo_2_O_4_	0.0071	-ln(C/C_0_) = 0.0071x − 0.05228	*R*^2^ = 0.95995
Ag@AgCl	0.00657	-ln(C/C_0_) = 0.00657x − 0.08943	*R*^2^ = 0.8517
0.3Ag@AgCl/ZnCo_2_O_4_	0.00239	-ln(C/C_0_) = 0.00239x + 0.03181	*R*^2^ = 0.9251
ZnCo_2_O_4_	0.00107	-ln(C/C_0_) = 0.00107x + 0.03186	*R*^2^ = 0.7338

The stability of catalyst is an important factor for its practical application. Figure 6d is the stability test results of 0.2Ag@AgCl/ZnCo_2_O_4_ recycled four times. It can be seen from Fig. 6d that the degradation effect of the catalyst has no obvious change after four recycles. The degradation rate of the sample decreases from 99.4 to 85%. The decrease of degradation rate may be due to the small amount of catalyst lost during each cycle. The loss of catalyst may be reduced by high-speed centrifugation during washing. In brief, the stability of 0.2Ag@AgCl/ZnCo_2_O_4_ is still very good if the experimental conditions are allowed without catalyst loss, so 0.2Ag@AgCl/ZnCo_2_O_4_ as a new type of visible photocatalyst has great value and potential for practical production.

**Fig. 7:**
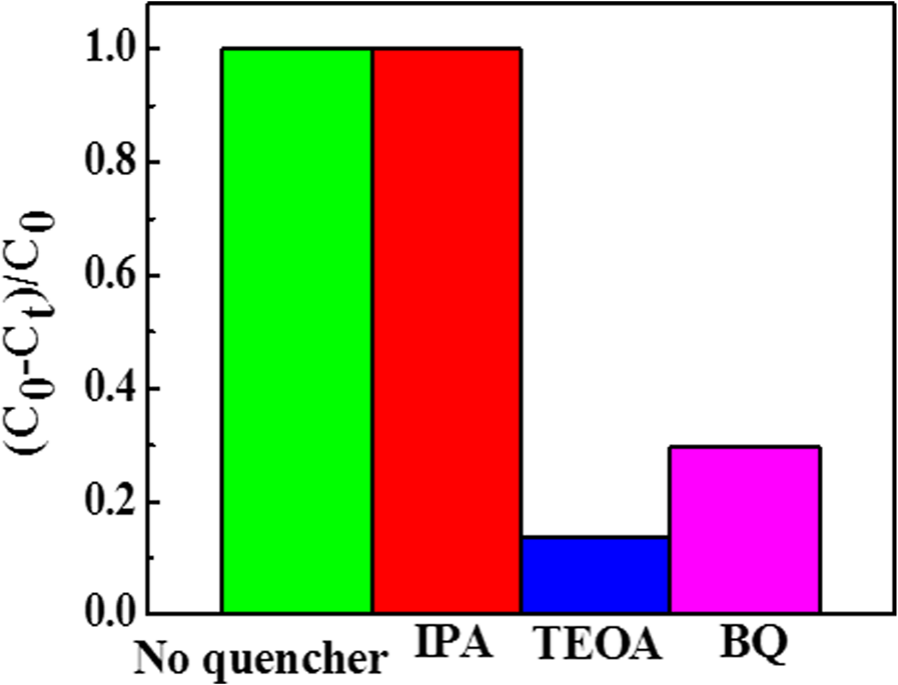
Trapping experiments of active species during the photocatalytic reaction

In order to understand the active factors in the Ag@AgCl/ZnCo_2_O_4_ photocatalytic degradation of RhB process, photocatalytic capture experiment were explored. Here, hydroxyl radicals (·OH), superoxide anions (O^·−^_2_), and holes (h^+^) are quenched by adding 1 mmol of isopropanol (IPA), p-benzoquinone (BQ), and triethanolamine (TEOA), respectively. Figure 7 shows the effect of capture of different active factors on the reaction rate in the process of photocatalytic reaction. It can be seen from the graph that the degradation rate of RhB is almost no less than that of RhB after adding 1 mmol IPA for 30 min. After adding BQ or TEOA, the degradation degree of RhB decreased greatly, especially when adding TEOA, the degradation rate was close to zero. Therefore, we can infer that the main active factors of Ag@AgCl/ZnCo_2_O_4_ photocatalyst are superoxide anion (O^·−^_2_) and hole (h^+^), not hydroxyl radical (·OH).

**Scheme 1:**
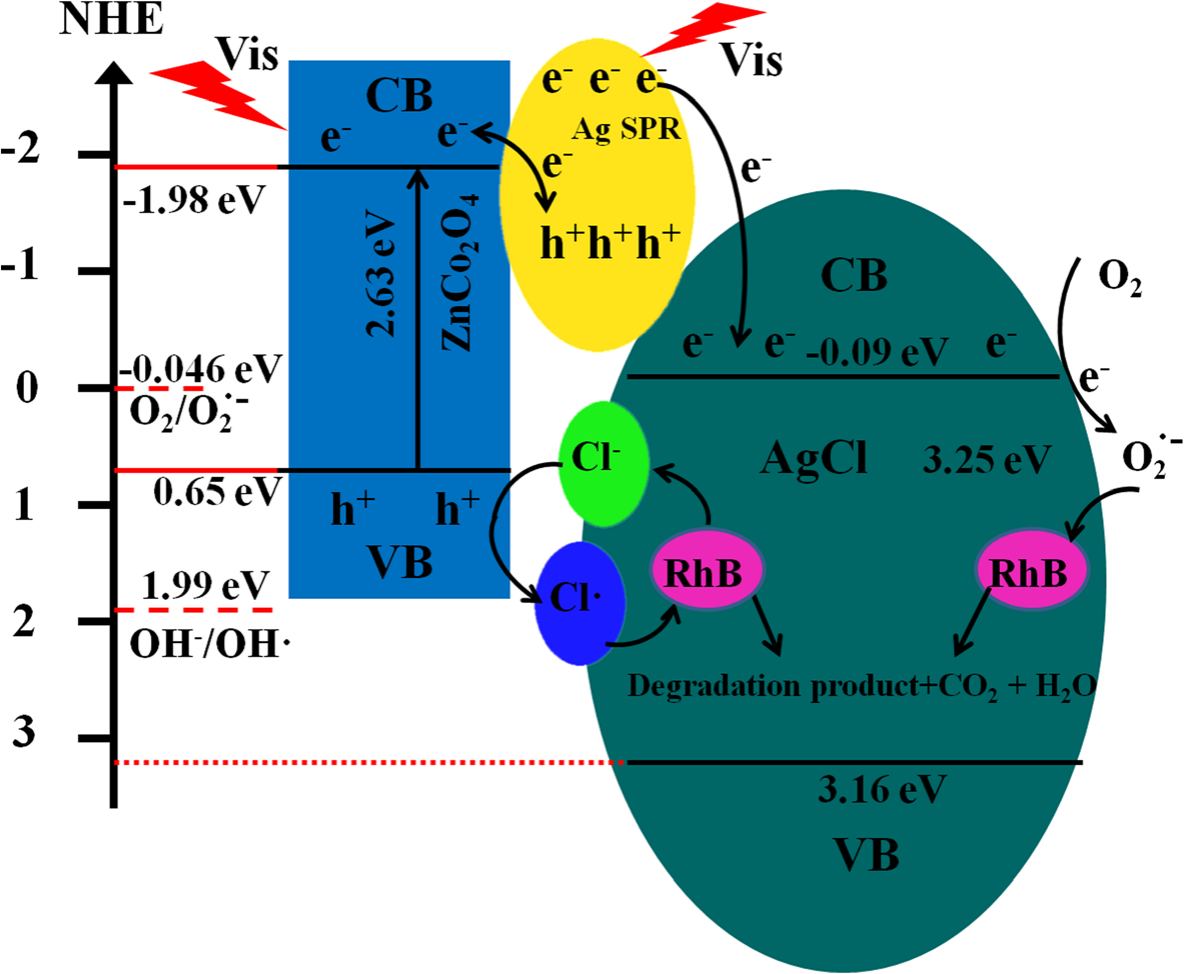
Schematic illustration of the photocatalytic mechanism of Ag@AgCl/ZnCo_2_O_4_ microspheres

Based on the experimental results and theoretical studies, we proposed a Z-scheme mechanism for photocatalytic degradation RhB. As shown in Scheme 1, Ag nanoparticles and ZnCo_2_O_4_ microspheres were excited to generate photogenerated electron-hole pairs under visible light irradiation. Electrons on Ag nanoparticles then are transferred to the conductive band of AgCl, and O_2_ adsorbed on the AgCl surface traps the electrons to produce O^·−^_2_, while photogenerated holes remain in the valence band of Ag nanoparticles. For ZnCo_2_O_4_, the relatively specific surface area is large and the adsorption capacity is strong, which can provide more adsorption sites for pollutants. The adsorbed pollutants can be transferred to the degradation center of the catalyst surface for pollutants degradation. The band gap of ZnCo_2_O_4_ is 2.63 eV. The conduction band and valence band energy levels of ZnCo_2_O_4_ are ca. − 1.98 eV and 0.65 eV (vs. NHE), respectively [31]. It shows that the photogenerated holes in the valence band of ZnCo_2_O_4_ are not directly involved in the degradation of the target pollutant, mainly because the energy of photogenerated holes is 0.65 eV (vs. NHE) lower than the reaction potential energy (E (OH^−^/·OH) = 1.99 eV (vs. NHE)). While the photogenerated electrons on the ZnCo_2_O_4_ conduction band transfer to Ag nanoparticles by the Schottky barrier and recombine with the photogenerated holes left on Ag nanoparticles. As the band gap width of AgCl is 3.25 eV, the conduction band and valence band energy levels of AgCl are ca. − 0.09 eV and 3.16 eV (vs. NHE) respectively, which cannot be excited with visible light; photogenerated electrons on Ag nanoparticles transfer into the AgCl conduction band and participate in the degradation of target pollutants, mainly because the energy of photogenerated electrons − 0.09 eV (vs. NHE) is more negative than the reaction potential energy at O_2_/O^·−^_2_ (E (O_2_/O^·−^_2_) = − 0.0 46 eV (vs. NHE)) [32]. The photogenerated holes on the valence band of ZnCo_2_O_4_ are transferred to the surface of AgCl and combined with Cl^−^ in AgCl to form Cl^·^ radicals. Cl^·^ radicals are strongly oxidizing and can degrade RhB effectively and mineralize into small inorganic molecules such as CO_2_ and H_2_O, and itself is reduced to Cl^−^. These Cl^−^ are then combined with Ag^+^ to regenerate AgCl to ensure the stability of the system. The results are consistent with the quenching experiment. In the photocatalytic degradation process of Ag@AgCl/ZnCo_2_O_4_, the main active factors are superoxide anion (O^·−^_2_) and hole (h^+^), not hydroxyl radical (·OH).

In summary, the formation, migration, and transformation of photoinspired electron-hole pairs and the final degradation pathways of pollutants during photocatalytic reaction are summarized as follows:

1. Generation of photoelectron hole pairs:

$$ \mathrm{Zn}{\mathrm{Co}}_2{\mathrm{O}}_4+\mathrm{hv}\to \mathrm{Zn}{\mathrm{Co}}_2{\mathrm{O}}_4\left({\mathrm{e}}^{-}\right)+\mathrm{Zn}{\mathrm{Co}}_2{\mathrm{O}}_4\left({\mathrm{h}}^{+}\right) $$

$$ \mathrm{Ag}+\mathrm{hv}\to \mathrm{Ag}\left({\mathrm{e}}^{-}\right)+\mathrm{Ag}\left({\mathrm{h}}^{+}\right) $$

(2) Migration and transformation of photogenerated hole electron pairs:

$$ \mathrm{Zn}{\mathrm{Co}}_2{\mathrm{O}}_4\left({\mathrm{e}}^{-}\right)+\mathrm{Ag}\left({\mathrm{h}}^{+}\right)\to \mathrm{Zn}{\mathrm{Co}}_2{\mathrm{O}}_4+\mathrm{Ag} $$

$$ \mathrm{Ag}\left({\mathrm{e}}^{-}\right)+\mathrm{AgCl}\to {\mathrm{O}}_2^{\cdotp -}+\mathrm{AgCl} $$

$$ \mathrm{Zn}{\mathrm{Co}}_2{\mathrm{O}}_4\left({\mathrm{h}}^{+}\right)+{\mathrm{Cl}}^{-}\to \mathrm{Zn}{\mathrm{Co}}_2{\mathrm{O}}_4+{\mathrm{Cl}}^0 $$

(3) Degradation of pollutants:

$$ {\mathrm{O}}_2^{\cdotp -}+\mathrm{RhB}\to \mathrm{Degradation}\ \mathrm{product}+{\mathrm{CO}}_2+{\mathrm{H}}_2\ \mathrm{O} $$

$$ {\mathrm{Cl}}^0+\mathrm{RhB}\to \mathrm{Degradation}\ \mathrm{product}+{\mathrm{CO}}_2+{\mathrm{H}}_2\ \mathrm{O}+{\mathrm{Cl}}^{-} $$

## Conclusions

In summary, the composite Ag@AgCl/ZnCo_2_O_4_ microspheres photocatalyst was prepared by a facile two-step method and characterized by a set of complementary structural and electronic characterization tools such as X-ray diffraction (XRD), scanning electron microscopy (SEM), energy dispersive X ray spectroscopy (EDX), transmission electron microscopy (TEM), high-resolution transmission electron microscopy (HR-TEM), selected area electron diffraction (SAED), X-ray photoelectron spectroscopy (XPS), UV-Vis diffuse reflectance spectroscopy (DRS), and Brunauer-Emmett-Teller (BET). Present results show that the composite photocatalyst has good crystal morphology, is highly crystalline, and the absorption intensity of Ag@AgCl/ZnCo_2_O_4_ composite photocatalyst in the whole spectrum range is higher than that of pure ZnCo_2_O_4_. The specific surface area of Ag@AgCl/ZnCo_2_O_4_ composite photocatalyst and the adsorption efficiency of RhB are found to increase as a result of Ag@AgCl deposition. In the degradation system of RhB, the photocatalytic degradation of pure ZnCo_2_O_4_ was the worst and the photocatalytic degradation rate for 120 min is found to have a very low value of 28%. The photocatalytic degradation rate of Ag@AgCl for 120 min is seen to be 86.3%. The results indicate that within 120 min, 99.4% photocatalytic degradation of 0.2Ag@AgCl/ZnCo_2_O_4_ takes place and RhB is found to be completely degraded. The reaction rate constant of 0.2Ag@AgCl/ZnCo_2_O_4_ composite photocatalyst is the highest showing a value of 0.01063 min^−1^, which is 1.6 times that of Ag@AgCl and 10 times of the minimum value of ZnCo_2_O_4_. In the reaction system of Ag@AgCl/ZnCo_2_O_4_, the main active factors of Ag@AgCl/ZnCo_2_O_4_ photocatalyst are found to be superoxide anion (O^·−^_2_) and hole (h^+^) and not hydroxyl radical (·OH). The photocatalytic mechanism of composite Ag@AgCl/ZnCo_2_O_4_ photocatalyst for the RhB degradation can be explained by a plasmonic Z-scheme photocatalytic mechanism, where the photogenerated electrons from the ZnCo_2_O_4_ conduction band at the contact interface of composite photocatalyst Ag@AgCl/ZnCo_2_O_4_ transfer to Ag nanoparticles by the Schottky barrier and recombine with photogenerated holes left on the Ag nanoparticles.

## Abbreviations

BET: Brunauer-Emmett-Teller
BQ: p-Benzoquinone
DRS: UV-Vis diffuse reflectance spectroscopy
EDX: Energy dispersive X ray spectrometer
HR-TEM: High-resolution transmission electron microscopy
IPA: Isopropanol
RhB: Rhodamine B
SAED: Selected area electron diffraction
SEM: Scanning electron microscopy
SPR: Surface plasmon resonance
TEM: Transmission electron microscopy
TEOA: Triethanolamine
XPS: X-ray photoelectron spectroscopy
XRD: X-ray diffraction

## References

[1] Wen B, Li Y, Chen C, Ma W, Zhao J (2010). Chem Eur J.

[2] Zeng P, Li J, Ye M, Zhuo K, Fang Z (2017). Chem Eur J.

[3] Zeng P, Wang X, Ming Y, Ma Q, Zhen F (2016). RSC Adv.

[4] Zhao Y, Bi M, Qian F, Zeng P, Chen M, Wang R, Liu Y, Ding Y, Fang Z (2018). ChemElectroChem.

[5] Kubacka A, Ga N, Fernã M, Colã N G (2016). Chem Rev.

[6] Hou D, Zhou W, Zhou K, Zhou Y, Zhong J, Yang L, Lu J, Li G, Chen S (2015). J Mater Chem A.

[7] Hou D, Zhou W, Liu X, Zhou K, Xie J, Li G, Chen S (2015). Electrochim Acta.

[8] Yang J, Wang D, Han H, Li C (2013). Acc Chem Res.

[9] Chen C, Ma W, Zhao J (2010). Chem Soc Rev.

[10] Ho W, Yu JC, Lee S (2006). Chem Commun.

[11] Chong MN, Jin B, Chow CW, Saint C (2010). Water Res.

[12] Zhou J, Cheng Y, Yu J (2011). J Photochem Photobiol A Chem.

[13] Xu Z, Zhang S, Gao F, Wen L, Yu Y, Li G (2018). Nanotechnology.

[14] Lin H (2011). Acta Phys -Chim Sin.

[15] Jia Z, Ren D, Wang Q, Zhu R (2013). Appl Surf Sci.

[16] Baird T, Campbell K, Holliman P, Hoyle RW, Stirling D, Williams BP, Morris M (1997). J Mater Chem.

[17] Kokane SB, Suryawanshi SR, Sasikala R, More MA, Sartale SD (2017). Mat Chem Phys.

[18] Guo H, Chen J, Weng W, Wang Q, Li S (2014). Chem Eng J.

[19] Goswami K, Ananthakrishnan R, Mandal S (2018). Mat Chem Phys.

[20] Wang X, Li S, Ma Y, Yu H, Yu J (2011). J.phys.chem.c.

[21] Li X, Fang S, Lei G, Han C, Ping Q, Liu W (2015). App Catal B Environ.

[22] Adhikari R, Gyawali G, Sekino T, Lee SW (2013). J Solid State Chem.

[23] Luo G, Jiang X, Li M, Shen Q, Zhang L, Yu H (2013). Appl Mater Interfaces.

[24] Fuku K, Hayashi R, Takakura S, Kamegawa T, Mori K, Yamashita H (2013). Angew Chem.

[25] Che H, Liu A, Zhang X, Mu J, Bai Y, Hou J (2015). Ceram Int.

[26] Hung TF, Mohamed SG, Shen CC, Tsai YQ, Chang WS, Liu RS (2013). Nanoscale.

[27] Moránlázaro JP, Lópezurías F, Muñozsandoval E, Blancoalonso O, Sancheztizapa M, Carreonalvarez A, Guillénbonilla H, Olveraamador ML, Guillénbonilla A, Rodríguezbetancourtt VM (2016) Sensors 16:2162–2176.

[28] Tachikawa T, Majima T (2010). Cheminform.

[29] Zhou Z, Long M, Cai W, Cai J (2012). J Mol Catal A Chem.

[30] Schuhl Y, Baussart H, Delobel R, Bras ML, Leroy JM, Gengembre L, Grimblot J (1983). Chemischer Informationsdienst.

[31] Chen L (2015) MSdiss, Dalian Polytechnic University, Tianjin, China

[32] Ye L, Liu J, Gong C, Tian L, Peng T, Zan L (2012). ACS Catal.

